# Vasculitis as an indicator of disease severity in familial Mediterranean fever

**DOI:** 10.3389/fimmu.2025.1506457

**Published:** 2025-08-12

**Authors:** Aviv Barzilai, Yarin Mash, Rotem Gershon, Elon Pras, Sharon Baum

**Affiliations:** ^1^ Department of Dermatology, Sheba Medical Centre, Ramat Gan, Israel; ^2^ Institute of Pathology, Sheba Medical Centre, Ramat Gan, Israel; ^3^ Faculty of Medical & Health Sciences, Tel Aviv University, Tel Aviv, Israel; ^4^ Department of Human Genetics, Sheba Medical Centre, Ramat Gan, Israel

**Keywords:** familial Mediterranean fever, vasculitis, superficial cutaneous vasculitis, immunoglobulin vasculitis/Henoch-Schonlein purpura, polyarteritis nodosa, Behçet disease, Pras severity score

## Abstract

**Introduction:**

Various types of vasculitides have been identified in patients with familial Mediterranean fever (FMF); however, FMF characteristic in patients who experience vasculitis during the disease course have not been described. This study aimed to describe the types of vasculitides in FMF and characterize the patients.

**Methods:**

This nested case-control study compared 27 patients with FMF (12 male) diagnosed with vasculitis with 100 patients (49 men) who did not develop vasculitis.

**Results:**

Most patients (25/27) developed vasculitis after FMF diagnosis. Four types of vasculitides were observed: cutaneous small vessel vasculitis (10 patients, 37%), Henoch–Schonlein purpura/immunoglobulin A vasculitis (8 patients, 30%), periarteritis nodosa (three patients, 11%), and Behçet disease (six patients, 22%). The vasculitis group was younger at FMF onset (6.6 [± 5.9] years vs. 16.2 [± 13.7] years, p < 0.002) and diagnosis (13.1 [± 13.1] years vs. 25.1 [± 17.92] years, p < 0.001). This group showed a higher frequency of homozygosity for the M694V mutation (73.9% vs. 29.4%, p < 0.001), had a more severe FMF (mean Pras severity score: 10.4 [± 2.6] vs. 7.3 [± 3.1], p < 0.001), required higher colchicine doses (1.96 [± 0.61] mg/d vs. 1.66 [± 0.65] mg/d, p < 0.025), and tended to show higher rates of colchicine resistance (29.6% vs. 12%, p = 0.053). However, vasculitis was not an independent factor influencing FMF severity.

**Conclusion:**

Patients with FMF and vasculitis are characterized by a more severe disease, likely due to factors other than vasculitis itself. Yet, its presence can serve as a clinical clue to disease severity.

## 1 Introduction

Familial Mediterranean fever (FMF), also known as paroxysmal polyserositis, is the most common hereditary autoinflammatory disease (1). Despite its global occurrence, it is prevalent in countries surrounding the Mediterranean Sea, especially Turkey and Israel, but also in other countries at the basin (1). In Israel, the FMF prevalence is 1–2 individuals per 1,000 population (2). The hallmarks of FMF are recurrent attacks characterized by an abrupt onset of fever and serosal inflammation, which manifest as abdominal and chest pain. Typically, the peritoneum, pleura, joints, and skin are involved. The attack frequency, duration, and severity vary, and > 80% of patients are symptomatic by 20 years of age (3). Secondary amyloidosis is a major complication of FMF that is associated with long-term morbidity and mortality (2, 4, 5), eventually leading to end-stage renal disease. The pathognomonic cutaneous manifestation of FMF is erysipelas-like erythema (ELE) (6, 7). Clinically, patients with a more severe disease are characterized by an early onset and early diagnosis, more frequent pleuritis, ELE, arthritis, and myalgia, resistance to colchicine and M694V mutations (see below) (8, 9). Considering its heterogeneous clinical expression, FMF is diagnosed based on clinical symptoms, ethnic origin, family history, and genetic testing.

FMF is presumed to be caused by the autosomal recessive inheritance of variants in the Mediterranean fever (*MEFV*) gene, located on the short arm of chromosome 16. The most common FMF-associated variant is the missense variant M694V in exon 10. Other common pathogenic *MEFV* variants include M680I, V726A, and M694I. These missense variants account for more than two-thirds of FMF cases in at-risk populations. The E148Q variant in exon 2 is associated with a milder form of FMF (10). Nevertheless, even among at-risk populations, approximately one-fourth of patients with clinical FMF carry either one or no variant (11). The expression of an FMF phenotype in individuals with a single *MEFV* variant suggests that a single-gene recessive model is insufficient to describe the complexity of FMF genetics, implying that modifier genes or epigenetic/environmental factors play a significant role in the disease development. Pyrin (marenostrin), the protein encoded by *MEFV* gene, acts as an inflammation-regulating factor by interacting with the inflammasome components. *MEFV* mutations result in a defective pyrin, leading to a consecutive activation of the inflammasome.

Patients with FMF, as with other monogenic autoinflammatory diseases, may present with vasculitis during the disease course (12). The mutated pyrin seems to interact poorly with inhibitors of inflammatory cascades, resulting in the production of interleukin-1β and nuclear factor-κB, eventually causing an inflammatory burst (13) and, importantly, contributing to vasculitis development in patients with FMF (14, 15). Although the types of vasculitides associated with FMF and associated comorbidities have been characterized (16–20), their relationship with the disease course and severity has not been widely described. Thus, this study aimed to characterize patients with FMF and vasculitis and elucidate whether they form a distinct subgroup of patients with FMF.

## 2 Materials and methods

### 2.1 Study design and patients

This nested case-control study was approved by the Institutional Review Board of Sheba Medical Centre (approval number: SMC 8401-21) and was conducted according to the tenets of the Declaration of Helsinki.

All patients were diagnosed, treated, and followed up in the Dermatology Department and FMF Outpatient Clinics at Sheba Medical Centre, a tertiary center in Israel, between 2009 and 2022. The inclusion criteria were (1) Diagnosis of FMF made at Sheba FMF clinic according to the (modified) Livneh criteria (21) or according to the Tel Hashomer criteria (22) (2) a minimum follow-up period of 3 months; and (3) an established clinical diagnosis of vasculitis. The various vasculitides were diagnosed by experience rheumatologists and FMF experts according to the accepted criteria at time of diagnosis which has a span of 13 years (2009-2022). Patients with an inconclusive diagnosis of either FMF or vasculitis, with missing data in their medical records, with a short follow-up period, or with incomplete records were excluded. Patients who were diagnosed with vasculitis were compared to sequentially selected patients who did not develop vasculitis. The diagnoses of FMF and vasculitis were identified from the institutional electronic medical records.

### 2.2 Data collection

The electronic medical files of the patients were reviewed to collect data on demographics, follow-up time, genetic testing, histopathological findings, clinical parameters (age at onset, age at FMF diagnosis, FMF severity score, frequency of FMF attacks, and the main sites involved), comorbidities, treatment administered, and treatment resistance. For patients with FMF diagnosed with vasculitis, data on the clinical type of vasculitis (palpable purpura-either isolated cutaneous leukocytoclastic vasculitis/cutaneous small vessel vasculitis[CSVV] or Henoch-Schonlein purpura [HSP]/IgA vasculitis [IgAV]; nodules-polyarteritis nodosa [PAN]/Behçet disease [BD]) and its temporal relationship to the FMF diagnosis were collected. Other investigated parameters of vasculitis included histopathological findings described in the pathological report, direct immunofluorescence (DIF) results, episode frequency, systemic manifestations, administered vasculitis treatments, and resistance to treatment.

### 2.3 Assessment

The patients were divided into two groups according to the comorbid occurrence of vasculitis. The vasculitis group was further subdivided according to the revised international Chapel Hill conference (ICHC) nomenclature of vasculitides (23) into those with systemic vasculitis (HSP/IgAV, PAN, and BD) or cutaneous small vessel vasculitis (CSVV) when only skin was involved. We did not use the term cutaneous leukocytoclastic vasculitis used in the ICHC as histology was available for only half of the patients. In brief, CSVV is defined as a single-organ, skin-isolated small vessel vasculitis or angiitis, often leukocytoclastic (LCV), without systemic vasculitis or glomerulonephritis. IgAV/HSP is an immune complex small vessel vasculitis with IgA1-dominant immune deposits. Glomerulonephritis indistinguishable from IgA nephropathy may occur. PAN is necrotizing arteritis of medium or small arteries without glomerulonephritis or vasculitis in arterioles, capillaries, or venules; and not associated with ANCA. BD is a various vessels vasculitis, and as such may involve many organs. However, kidneys are rarely involved. All collected parameters were compared between the groups. FMF severity in both groups was assessed using the Pras severity scoring system (19, 24), **Supplementary Table S1**). This scoring system is a short, widely used 19-point score comprising six parameters (age at disease onset, number of attacks per month, presence of arthritis, erysipelas such as erythema, amyloidosis, and daily colchicine dosage) that are each assigned numerical scores between 1 and 4. The overall FMF severity is the sum of the scores for each parameter, in which scores of 3–5, 6–8, and ≥ 9 indicate mild, intermediate, and severe disease, respectively.

### 2.4 Statistical analysis

Categorical variables are described as N (%) and compared using the chi-square test. Continuous variables are described as means ± standard deviation (SD) and compared using Student’s t-test. In cases where one of the cells in a contingency table contained five or fewer samples, Fisher’s exact test was used. All statistical analyses were conducted using R version 3.6.3 with the R base and TableOne packages. All tests were performed at a 95% confidence level (significance level of 0.05) with an 80% statistical power.

## 3 Results

### 3.1 Epidemiological, clinical, and histopathological features of FMF-associated vasculitis

This study included 27 patients with FMF and vasculitis (12 men and 15 women, mean age at vasculitis diagnosis 38.8 ± 20.6 [range, 9–79] years, 4 of them children (<12 years old), 1 adolescent (<18 years old)). The patients’ characteristics are provided in **Table 1**. Overall, 25 patients (92.6%) were diagnosed with FMF before vasculitis. In total, 13 of the 27 patients had skin biopsies showing morphological findings, DIF findings, or both of vasculitis. Immunoglobulin A (IgA) nephropathy was diagnosed in one patient who underwent a kidney biopsy. Further classification of vasculitis based on the clinicopathological correlations resulted in four major subtypes: (1) CSVV (n = 10 patients, 37%), (2) HSP/IgAV (n = 7 patients, 25.9%) and protracted febrile myalgia (PFM, n = 1 patient, included further on in the HSP group), (3) PAN (n = 3 patients, 11.1%), and (4) BD-associated vasculitis (n = 6 patients, 22.2%). Palpable purpura was observed in 20 patients: 18 with CSVV/HSP but also in 2 patients with PAN and BD.

**Table 1:** Characteristics of patients with FMF-associated vasculitis (n = 27).

Variable	Value
Sex (female)	15 (55.6)
Age at vasculitis diagnosis (years), mean ± SD; range	38.78 ± 20.6; 9–79
FMF diagnosis prior to vasculitis (number of patients)	25 (92.6)
Histopathological findings	
Biopsy of vasculitis	11 (40.7)
Direct immunofluorescence (DIF) of vasculitis	5 (18.5)
Vasculitis classification	
HSP/IgAV*	8 (29.6)
PAN	3 (11.1)
BD	6 (22.2)
CSVV	10 (37)
Vasculitis episodes	
Ongoing	3 (11.1)
Single episode	10 (37)
Two episodes	3 (11.1)
Multiple episodes	11 (40.8)
Specific Vasculitis treatment	22 (81.5)
Corticosteroids	21 (77.8)
NSAIDS	4 (14.8)
Cyclosporine	4 (14.8)
Methotrexate	4 (14.8)
Biologic treatment**	5 (18.5)
Response to treatment	16 (72.7)

Data are expressed as n (%) or the mean ± S.D. unless otherwise specified.

BD, Behçet’s disease; CSVV, cutaneous small vessel vasculitis: DIF, direct immunofluorescence; PAN, polyarteritis nodosa; NSAIDS, nonsteroidal anti-inflammatory drugs.

*including protracted fibromyalgia -1 patient.

**given for the FMF.

During the follow up period of 6 months to 13 years, among the 27 patients with vasculitis, 11 (40.8%), 3 (11.1%), and 10 (37%) experienced multiple, two, and a single flares of their FMF, respectively. Three (11.1%) patients had a single ongoing episode during their last clinical visit. The two most commonly affected systems during vasculitis episodes were the gastrointestinal and renal (7 patients each, 25.9%) systems. Renal involvement was evident in 6 patients with IgAV as glumerolonephritis findings, and in one patient with PAN that developed renal failure with fluid retention. GI involvement was diagnosed in 5 patients with IgAV and in 2 patients with BD, in all the clinical manifestation was either abdominal pains or bloody diarrhea or both. Nervous system (central nervous system in 4 patients with BD and peripheral nervous system in 2, HSP and PAN) and joint involvement (as part of the vasculitis) were observed in six patients (22.2%) each. A total of 22 patients (81.5%) received specific vasculitis treatment, with a response rate of 72.7%. Systemic corticosteroids were the most common treatment (21/22 patients, 95.5%), followed by nonsteroidal anti-inflammatory drugs, cyclosporine, methotrexate. Five patients received anti interleukin 1 therapies (anakinra, canakinumab) due their FMF. Yet, in all these patients the vasculitis appeared prior to the biologic initiation and did not recur afterwards. Compared with patients with systemic vasculitis, those with CSSV had a more severe FMF (mean severity score: 9.5 ± 2.1 vs. 11.8 ± 2.8, *p* = 0.025). These patients tended to have a higher rate of ELE.

### 3.2 Comparison between patients with FMF associated with and without vasculitis

A total of 100 patients (49 men and 51 women) with FMF who did not develop vasculitis were included. **Table 2** summarizes the demographic, clinical, and genetic features of the patients with FMF with and without vasculitis.

**Table 2:** Characteristics of patients with FMF associated with and without vasculitis.

Variable	FMF patients with vasculitis (n = 27)	FMF patients without vasculitis (n = 100)	P value
Sex (female)	15 (55.6)	51 (51.0)	0.839
Age at FMF onset (years)	6.58 ± 5.9	16.21 ± 13.7	**0.001**
Age at FMF diagnosis (years)	13.12 ± 13.1	25.11 ± 17.9	**0.002**
FMF duration (years)	36.31 ± 20.2	26.59 ± 17	**0.014**
Delay in FMF diagnosis (years)	6.54 ± 10.6	8.89 ± 12.9	0.392
Familial history of FMF	20 (76.9)	62 (62)	0.245
Ethnicity			
Arab	1 (3.7)	2 (2)	0.176
North African	20 (74.1)	50 (50)	
Middle Eastern	4 (14.8)	23 (23)	
East European	1 (3.7)	16 (16)	
Mixed ethnicity	1 (3.7)	9 (9)	
Genetics			
Genetic test not available	4 (14.8)	6 (6)	
Genetic test negative for *MEFV*	0 (0)	9 (9)	
Genetic test positive for *MEFV*	23 (100)	85 (91)	0.202
Genotype			
M694V/ M694V	17 (73.9)	25 (29.4)	**0.001**
V726A/ V726A	0 (0)	1 (1.2)	
M694V/-	3 (13)	30 (35.3)	
V726A/-	0 (0)	5 (5.9)	
E148Q/-	1 (4.3)	0 (0.0)	
M694V/ V726A	1 (4.3)	6 (7.1)	
M694V/ E148Q	0 (0)	11 (12.9)	
E148Q/ V726A	1 (4.3)	4 (4.7)	
A744S/ V726A	0 (0)	2 (2.4)	
K695R/ V726A	0 (0)	1 (1.2)	
Inflammatory skin disease	5 (18.5)	7 (7)	0.148
Papulosquamous	2 (7.4)	0 (0)	0.061
Autoimmune	2 (7.4)	3 (3)	0.151
Urticaria	0 (0)	1 (1)	1
Comorbidities	15 (55.6)	47 (47)	0.567
Cardiovascular	11 (40.7)	16 (16)	**0.012**
Autoimmune (classic, MHC class II associated)	5 (18.5)	7(7)	0.12
Endocrine	3 (11.1)	10 (10)	1
Metabolic	6 (22.2)	22 (22)	1
Neurologic	7 (25.9)	8 (8)	**0.026**
Hematologic	8 (29.6)	3 (3)	**<0.001**
Malignancy	4 (14.8)	6 (6)	0.269
Pulmonary	7 (25.9)	6 (6)	**0.008**
FMF clinical manifestations			
Arthritis	27 (100)	62 (62)	**<0.001**
Carditis	1 (3.7)	3 (3)	1
Pleuritis	21 (77.8)	39 (39)	**0.001**
Peritonitis	27 (100)	83 (83)	**0.047**
Erysipelas-like erythema (ELE)	9 (33.3)	11 (11)	**0.011**
Number of attacks per month	1.62 ± 1.8	0.69 ± 1.4	0.005
Colchicine treatment	27 (100)	99 (99)	1
Colchicine dose (g/day)	1.96 ± 0.61	1.66 ± 0.65	**0.025**
Colchicine resistance	8 (29.6)	12 (12)	0.053
Amyloidosis	3 (11.1)	6 (6)	0.147
FMF severity score	10.37 ± 2.6	7.32 ± 3.1	**<0.001**
FMF severity classification			
Mild disease	1 (3.7)	32 (32)	**0.001**
Intermediate disease	6 (22.2)	31 (31)	
Severe disease	20 (74.1)	37 (37)	

Data are expressed as n (%) or the mean ± S.D.

FMF, familial Mediterranean fever; SD, standard deviation; *MEFV*: Mediterranean Fever (gene); ELE, erysipelas-like erythema.Statistically significant values (p<0.05) are marked in bold.

#### 3.2.1 Epidemiology

In both groups, the most common ethnicity was of North African origin (55.1%), followed by Middle Eastern origin (21.3%). The rates of family history of FMF were similar between the two groups. The vasculitis group was younger at FMF diagnosis (13.1 [± 13.1] years vs. 25.1 [± 17.9] years) *p* < 0.001), had earlier disease onset (6.6 [± 5.9] years vs. 16.2 [± 13.7] years, *p* < 0.002), had a longer FMF duration from the time of onset (36.3 [± 20.2] years vs. 26.6 [± 17] years, *p* < 0.014), and tended to have a shorter interval between FMF onset and clinical diagnosis (6.5 [± 10.6] years vs. 8.9 [± 12.9] years, *p* = 0.39).

#### 3.2.2 Genetics

Genotype analysis of the *MEFV* gene was available in 117 patients. *MEFV* variants were observed in 108 of them (91%), with comparable prevalence rates between the groups (23/23, 100%, and 85/94, 91%, respectively). All 9 variants of negative patients did not experienced vasculitis. Overall, 43 patients (39.8%) were homozygous, and 65 patients (60.2%) were heterozygous for at least one *MEFV* variant, among whom 26/65 (24.1%) showed 2 different mutations in the *MEFV* gene, i.e., a compound heterozygous genotype. Further stratification of distinct genotypes in each group is provided in **Table 2**. Among the five *MEFV* variants investigated, M694V was predominant in both groups. At least one allele with the M694V variant was observed in 23/23 100%) patients in the vasculitis group and in 72/94 (76.5%) patients in the no-vasculitis group (*p* = 0.007).

Homozygosity for the M694V variant was more common in the vasculitis group (17/23 [73.9%] vs. 25/85 [29.4%], *p* < 0.001). In the non-vasculitis group, heterozygosity for the M694V variant was the most common genotype, detected in 30 patients (35.3%). In contrast, heterozygosity for the same variant was observed in only three patients (13%) in the vasculitis group (*p* < 0.001). Heterozygosity and compound heterozygosity were more common in the no-vasculitis group (59/85 [69.4%] vs. 6/23 [26%], *p* < 0.001).

#### 3.2.3 Clinical characteristics and treatment

The clinical characteristics of FMF and the therapeutic modalities for the disease in both groups are provided in **Table 2**. Significantly higher rates of arthritis (100%, 27 patients), pleuritis (77.8%, 21 patients), peritonitis (100%, 27 patients), and ELE (33.3%, 9 patients) were observed in the vasculitis group. This group exhibited a significantly higher frequency of FMF attacks (mean: 1.6 ± 1.8 attacks vs. 0.7 ± 1.4 attacks, *p* = 0.005). While amyloidosis was more common in the vasculitis group (3 [11.1%] vs. 6 [6%]), the difference was not significant. This group showed higher rates of cardiovascular, pulmonary, neurological, hematological, but not classical, MHC class II related, autoimmune comorbidities. All patients except for one were treated with colchicine. The daily colchicine dose tended to be higher in the vasculitis group (1.96 [± 0.61] mg/day vs. 1.66 [± 0.65] mg/day, *p* < 0.025). Colchicine resistance was more common in the vasculitis group than in the no-vasculitis group (8/27 [29.6%] vs. 12/100 [12%]), with a borderline significant difference (*p* = 0.053).

#### 3.2.4 Severity of familial Mediterranean fever in patients with and without vasculitis

The FMF severity scores are summarized in **Table 2**. The mean was higher in the vasculitis group than in the no-vasculitis group (10.4 [± 2.6] vs. 7.3 [± 3.1], *p* < 0.001). Overall, 20/27 (74.1%) patients in the vasculitis group were classified as having severe disease, compared with only 37/100 patients in the no-vasculitis group (37%). Although vasculitis, not mentioned in the PRAS severity score, was associated with a more severe disease in our cohort, it was not an independent influencing factor of severe disease in the multivariate analysis (*p* = 0.63).

## 4 Discussion

The results of this nested-case control retrospective study demonstrated that among patients with FMF and vasculitis, the diagnosis of FMF generally (92% of cases) preceded that of vasculitis and that slightly more than half of the patients with FMF and vasculitis experienced more than one episode. Four major clinical subtypes of vasculitis were identified: CSVV, HSP/IgAV (including PFM), PAN, and BD-associated vasculitis. Most importantly, this is the first comparative study of patients with FMF according to the occurrence of vasculitis.

Previous studies have focused on three types of vasculitides with systemic manifestations in patients with FMF, namely, HSP/IgAV, PAN, and BD (14, 16, 17), with HSP being the most frequent. To date, CSVV alone (without systemic involvement) as a distinct type of vasculitides in FMF has not been described. In this study, most of the patients had CSVV; unexpectedly, these patients had higher FMF severity scores, which merits further clarification. Furthermore, most of our patients with vasculitis (20/27, 74%) presented clinically as palpable purpura, irrespective of the presence of systemic vasculitis. Moreover, because CSVV and ELE are neutrophilic dermatoses, CSVV in this setting may represent the same process as ELE, an established severity criterion.

In contrast to previous reports (16) describing a single episode of vasculitis, especially for IgAV, our study findings included a high rate of vasculitis manifesting in two or more episodes among patients with FMF (51.8%). However, the response rate to drugs commonly used in vasculitides or to biologic therapy for FMF, initiated after vasculitis diagnosis, was relatively satisfactory, with a response rate of 72.7%.

Our characterization of patients with FMF-associated vasculitis revealed that these patients formed a distinct group with a unique epidemiology, clinical course, genetics, and disease severity. We observed FMF onset and clinical diagnosis at an earlier age and an overall prolonged disease duration compared with patients with FMF without vasculitis. More patients with vasculitis were of North African origin and showed homozygosity for the M694V *MEFV* variant. The significant difference in the rate of homozygosity is consistent with previous findings on FMF-associated HSP, PAN, and BD (25–27). Furthermore, the presence of two M694V homozygosity seems to confer a significant risk for vasculitis. These findings, along with the clinical features described here, raise the previously discussed issue of whether vasculitis, especially HSP/IgAV and BD, are inherent aspects of the FMF disease itself and not a mere secondary comorbidity.

The patients with FMF and vasculitis in our study had a distinctive clinical course that encompassed different aspects. In addition to an earlier age at FMF onset, an earlier age at FMF diagnosis, and an overall longer disease duration, these patients showed a higher frequency of attacks and higher rates of arthritis (100% vs. 62%), pleuritis (77.8% vs. 39%), and peritonitis (100% vs. 83%) compared with patients without vasculitis. ELE was more common among patients with FMF-associated vasculitis (33.3% vs. 11%). Regarding treatment, dosage, and response, patients with FMF-associated vasculitis required higher daily doses of colchicine, and more patients with vasculitis showed colchicine resistance, although the difference was only borderline significant. Consequently, these patients experienced a more severe disease course. Evaluation of disease severity according to the severity scoring system described by Pras et al. (24) demonstrated a higher mean severity score and a higher proportion of patients with severe disease among patients with FMF and vasculitis. These findings are in line with previous reports and in certain aspects broaden them. Miray et al. assessed the influence of concomitant diseases on FMF severity in 494 children (18). They found that patients with concomitant diseases, especially juvenile idiopathic arthritis, experienced a more severe FMF. In this study, patients with HSP did not experience a more severe disease. In another study (19), it was confirmed that carrying *M694V* mutation is associated with a more severe FMF and with comorbidities, juvenile idiopathic arthritis and HSP being the most common of them. In a recent study, also describing children with FMF (20), comorbid patients exhibited characteristics similar to those found in patients with vasculitis (most of them adults) in our cohort. Yet, in both studies (19, 20), stratification of disease severity according to the various comorbidities is not described.

Our study shows that vasculitis tends to occur in patients with a more aggressive FMF, and therefore these patients shared the epidemiological, clinical, and genetic characteristics distinctive for severe FMF (8, 9). Furthermore, the clinical picture in this group of patients will also be complicated by the vasculitis itself. One pathogenic consideration might be that the pro-inflammatory phenotype of (severe) FMF may contribute to the development of vasculitides, by lowering the threshold for inflammation to become apparent.

Comorbidities such as cardiovascular, hematological, neurological, and pulmonary diseases were more common in patients with FMF and vasculitis. Although vasculitis itself, mainly BD, can explain the higher rate of neurological diseases among patients with vasculitis, a direct relationship might not exist for the other comorbidities. Despite the unclear risk of cardiovascular disease (28), the more severe disease in patients with FMF-associated vasculitis, which indicates more inflammation, may contribute to the development of other comorbidities in these patients, particularly cardiovascular diseases (29).

Our study is limited by its retrospective nested case-control design. Additionally, the diagnosis of vasculitis was based on clinical assessment without supporting biopsy in approximately half of the patients, and the sample size was relatively small, possibly contributing to the inability to show that vasculitis has an independent role in FMF severity. It is also important to note that most of our vasculitides comorbid patients were adults. Further studies with more participants are required to confirm our results.

In conclusion, unlike FMF patients who did not experience vasculitis, patients with FMF who develop vasculitis exhibit multiple epidemiological, genetic, and clinical characteristics associated a more severe FMF and a higher frequency of severe comorbidities. Therefore, this nouvelle observation may serve as a clinical marker to identify severe FMF patients, a unique, distinctive group of patients, and improve patients’ management. Our study further emphasizes that FMF and vasculitis remain inextricably associated, and further clinical and molecular studies are required to clarify this association.

## Data Availability

The raw data supporting the conclusions of this article will be made available by the authors, without undue reservation.
