# Supplementary material for: Vasculitis as an indicator of disease severity in familial Mediterranean fever
Source: Front Immunol. 2025 Aug 12;16:1506457. doi: 10.3389/fimmu.2025.1506457 (PMC12378754; doi:10.3389/fimmu.2025.1506457)
Supplement: Supplementary file 1 [file Table1.docx]

**Supplementary Table S1: Severity Scoring System by Pras et al^24^**

| Score | Features | Parameter |
| --- | --- | --- |
| 0  1  2  3  4 | 31<  21-31  11-20  6-10  6> | Age of onset (years) |
| 1  2  3 | 1>  1-2  2< | Number of attacks per month |
| 2  3 | Acute  Protracted | Arthritis |
| 2 |  | Erysipelas-like erythema |
| 3 |  | Amyloidosis |
| 1  2  3  4 | 1  1.5  2  2< | Colchicine dose (mg/day) |

The overall severity of a disease in a given patient is the sum of the scores for each parameter. A score of 3 to 5 is considered to reflect mild disease, 6 to 8 intermediate disease, and $\geq$ 9 severe disease.
